# Variables associated with physical fighting among US high-school students

**DOI:** 10.1186/1745-0179-4-16

**Published:** 2008-05-29

**Authors:** Emmanuel Rudatsikira, Adamson S Muula, Seter Siziya

**Affiliations:** 1 Departments of Global Health, Epidemiology and Biostatistics, School of Public Health, Loma Linda University, Loma Linda, California, USA; 2 Department of Community Health, University of Malawi, College of Medicine, Blantyre, Malawi; 3 Department of Community Medicine, University of Zambia, School of Medicine, Lusaka, Zambia

## Abstract

**Background:**

Violence among adolescents is an important public health problem in the United States. This study was conducted to estimate the prevalence of having been engaged in physical fighting on school property and associated factors of the behavior among school-going adolescents in the United States.

**Methods:**

This study was based on secondary analysis of the United States Youth Risk Behavior Survey (YRBS) conducted in 2005. The sampling frame included all private and public school in the country, stratified by region and urbanicity based on the US census bureau data. Frequencies and proportions were obtained for the outcome and explanatory variables. Logistic regression analysis was used to estimate the level of association between explanatory variables and the outcome (having been involved in a physical fight).

**Results:**

Of the 13,857 respondents, 13.5% (18.2% for males and 8.8% for females) reported physical fighting onschool property in the last 12 months to the survey. Males were more likely to have been in a physical fight than females (OR = 2.23; 95% CI [1.89, 2.63]). Respondents aged 17 years or older were less likely to report physical fighting than those who were 14 years or younger. Compared to Whites, American Indians or Alaska Natives, Blacks, Native Hawaii or other Pacific Islanders, and Hispanics were more likely to report physical fighting on school property (OR = 2.11; 95% CI [1.22, 3.66], OR = 1.72; 95% CI [1.42, 2.0], OR = 2.18; 95% CI [1.01, 4.79], and OR = 1.74; 95% CI [1.41, 2.16] respectively). Physical fighting on school property was also positively associated with cigarette smoking (OR = 1.70; 95% CI [1.37, 2.10]), drinking alcohol (OR = 1.45; 95% CI [1.20, 1.76]), use of illegal drugs (OR = 1.73; 95% CI [1.42, 2.12]), having had property stolen or deliberately damaged on school property (OR = 2.06; 95% CI [1.74, 2.44]), having been threatened or injured with a weapon on school property (OR = 2.63; 95% CI [2.06, 3.34]), and playing videogame three or more hours a day (OR = 1.29; 95% CI [1.07, 1.56]).

**Conclusion:**

These findings suggest that physical fighting among US High School students is widespread and positively associated with victimization (having been threatened or injured with a weapon on school property) and other risky behaviors such as smoking, alcohol and drugs use. Intervention programs to prevent/control those risky behaviors as well as further attention on the association between physical fighting and victimization at school through longitudinal research are warranted.

## Background

Interpersonal violence is a major cause of morbidity and mortality among adolescents and young adults in the United States [1,2]. Violent deaths in schools are one severe consequence of school-based interpersonal violence. Kachur et al have estimated a rate of 0.09 school-associated violent deaths per 100 000 student-years between the period 1992 to 1994 in the United States [3]. Pickett et al, have reported that involvement in adolescent fighting in North America and Europe varied across countries, ranging from 37% to 69% for boys and 13% to 32% of the girls [4]. In many of these circumstances, adolescents most often reported fighting with friends or relatives. Recent studies from the developing countries have also shown that physical fighting among school going adolescents is prevalent. Rudatsikira et al have reported that 50.6% of adolescents had engaged in a physical fighting within the past 12 months prior to the survey in Namibia, southern Africa [5].

While deaths are the most severe consequences of interpersonal violence, the effects of school violence include: interference with the normal learning activities of students; weapon carrying for defensive purposes on school grounds; serious injury and attendant medical costs and absenteeism from school [6-8]. The Centers for Disease Control and Prevention (CDC) have reported that adolescents who felt unsafe at school were likely to be absent [9].

Previous studies have reported on factors that are associated with having engaged in a physical fight among in-school adolescents. Kodjo et al, using data from the United States National Longitudinal Study of Adolescent Health (Add Health) of 1994–1995, a school-based, nationally representative survey of 6504 7th to 12th graders have studied the association of being involved in a fight and drug use [10]. The aim of this study was to estimate the prevalence of having engaged in physical fighting on school property and associated factors of the behavior among school-going adolescents in the United States.

## Methods

The primary sampling United States Youth Risk Behavior Survey (YRBS) of 2005 comprised all schools (public and private) with students in at least one of grades 9–12 in the 50 states and the District of Columbia. The sampling frame was obtained from the Quality Education Data (QED), Inc. database [11]. This database is one of the most comprehensive on the status of all schools in the United States. It includes information on both public and private schools and the most recent data from the Common Core of Data from the National Center for Education Statistics [12].

A three-stage cluster sample design was used to obtain a nationally representative sample of students in grades 9–12. In the first-stage of sampling, 1,261 primary sampling units (PSUs), consisting of counties, subareas of large counties, or groups of smaller, adjacent counties were obtained. The 1,261 PSUs were categorized into 16 strata according to their metropolitan statistical area (MSA) status. From these 1,261 PSUs, 57 were selected with probability proportional to overall school enrollment size for the PSU. In the second stage of sampling, a total of 203 schools with any of grades 9–12 were selected with probability proportional to school enrollment size. The third stage of sampling consisted of randomly selecting, in each chosen school and in each of grades 9–12, one or two classrooms from either a required subject or a required period (e.g., homeroom or second period).

All students in selected classes were eligible to participate. There was no replacement of schools, classes, or students that refused to participate. Black and Hispanic students were oversampled in order to allow for stratified analysis of these groups. The sampling of these groups provided that: 1) larger sampling rates were used to select PSUs that are in high-black and high-Hispanic strata; 2) a modified measure of size was used that increased the probability of selecting schools with a disproportionately high minority enrollment; and 3) two classes per grade, rather than one, were selected in schools with a high minority enrollment. A weight based on student sex, race/ethnicity, and grade level was applied to each record to adjust for school and student non-response and oversampling of black and Hispanic students. The overall weights were scaled so that the weighted count of students equals the total sample size, and the weighted proportions of students in each grade match the national population proportions. For the 2005 national YRBS, 13,953 questionnaires were completed in 159 schools. The school response rate was 78%, and the student response rate was 86%. The school response rate multiplied by the student response rate produced an overall response rate of 67%. CDC's Institutional Review Board granted clearance for the national YRBS.

### Data Collection and Questionnaire design

Study participants completed the questionnaires anonymously within a class period. Student completed the questionnaires on computer scannable booklet or answer sheet.

Local parental permission procedures were followed before questionnaire administration. These procedures differed from state to state and from school district to school district.

The core questionnaire had 87 questions. States and cities were free to add or delete any questions for whatever reasons. Information about the reliability of the core questionnaire is published elsewhere [13].

To assess physical fighting, participants were asked the following question: "During the past 12 months, how many times were you in a physical fight on school property? (0 times, 1 time, 2 or 3 times, 4 or 5 times, 6 or 7 times, 8 or 9 times, 10 or 11 times, 12 or more times). During the analysis, in both Univariate and Multivariate, we recorded "0 times" as "no" and "1 or more times" as "yes". Current cigarette smoking meant having smoked cigarettes one or more days in the last 30 days. Alcohol use was defined as having had at least one drink of alcohol one day or more in the last 30 days while illicit drug use meant having ever used any of the following: marijuana, any form of cocaine, glue, heroin, methamphetamines, ecstasy, and steroid pills or shots.

### Data analysis

Data analysis was only conducted for complete questionnaires. Our analysis is restricted to students in high school (elementary school excluded). As per requirement of the Office of Management and Budget of the US government, race/ethnicity categorization followed from the following questions and responses:

from two question: 1) "Are you Hispanic or Latino?" (options were "yes" or "no"), and 2) "What is your race?" (options were "American Indian or Alaska Native," "Asian," "Black or African American," "Native Hawaiian or Other Pacific Islander," or "White"). For the second question, students could select more than one response option. For this report, students were classified as "Hispanic" if they answered "yes" to the first question, regardless of how they answered the second question. Students were classified as "Black" if they answered "no" to the first question and selected only "Black or African American" to the second question. Students were classified as "White" if they answered "no" to the first question and selected only "White" to the second question. Students were classified as "other" if they answered "no" to the first question and selected "American Indian or Alaska Native," "Asian," and/or "Native Hawaiian or Other Pacific Islander" or selected more than one response to the second question.

For the 2005 state and local YRBS, race/ethnicity was computed from one question: "How do you describe yourself?" (response options were "American Indian or Alaska Native," "Asian," "Black or African American," "Hispanic or Latino," "Native Hawaiian or Other Pacific Islander," or "White"). Students could select more than one response option. For this report, students were classified as "Hispanic" if they selected "Hispanic or Latino" only or if they selected "Hispanic or Latino" plus any other response option. Students were classified as "Black" if they selected "Black or African-American" only. Students were classified as "White" if they selected "White" only. Students were classified as "other" in the recoding if they selected "American Indian or Alaska Native" only, "Asian" only, and/or "Native Hawaiian or Other Pacific Islander" only or multiple response options except "Hispanic or Latino."

Data were analyzed using SAS software version 9.0.3 (SAS, Cary, North Carolina, United States). We obtained frequencies and proportions of relevant variables. We also assess associations using multivariate logistic regression analysis with having engaging in a physical fight as the outcome. The variables assessed for association were those that have previously been reported in previous studies [14,15]. Bivariate analysis results report unadjusted effect estimates while multivariate analysis reports effect estimates following control of co-variates.

## Results

Table 1 presents selected characteristics of the study population of 13,857 US High School students (median age 15 years). Most of the sample was male (51.9%), White (67.8%), non cigarette smokers and non alcohol users. Overall 13.5% (18.2% males and 8.8% females) of the respondents reported having been in a physical fight on school property in the last 12 months to the survey.

**Table 1:** Characteristics of the study population of United States High School students in the Youth Risk Behavior Survey 2005

Characteristics	Total	Males	Females
Ethnicity			
Age (years)	n (%)	n (%)	n (%)
All	13857 (100)	7193 (51.9)	6664 (48.1)
≤ 14	1221 (8.8)	701 (9.8)	520 (7.8)
15	3170 (23.9)	1720 (23.9)	1450 (21.6)
16	3535 (25.5)	1817 (25.3)	1718 (25.8)
17	3661 (26.4)	1881 (26.2)	1780 (26.7)
18+	2270 (16.4)	1074 (14.8)	1196 (18.0)
Ethnicity			
White	6117 (50.5)	3015 (51.7)	3102 (49.3)
American Indian or Alaska Native	144 (1.2)	86 (1.6)	58 (0.9)
Asian	366 (3.1)	366 (3.2)	179 (2.8)
Blacks	3343 (27.6)	1574 (27.0)	1768 (28.1)
Native Hawaii or other Pacific Islander	90 (0.7)	44 (0.7)	46 (0.7)
Hispanics	2063 (17.3)	920 (15.8)	1143 (18.2)
Cigarette smoking in the last 30 days			
No	10374 (77.0)	4808 (77.0)	5566 (77.0)
Yes	2856 (23.0)	1490 (23.0)	1366 (23.0)
Alcohol use in the last 30 days			
No	7434 (56.7)	3428 (56.2)	4006 (57.2)
Yes	5751 (43.3)	2841 (43.8)	2910 (42.8)
Ever used illegal drugs			
No	7225 (54.0)	3212 (54.3)	4013 (57.8)
Yes	6228 (46.0)	3210 (45.7)	3018 (42.2)
Property stolen or deliberately damaged on school property in the last 12 months			
No	9699 (70.3)	4538 (68.6)	5161 (72.0)
Yes	41.32 (29.7)	2105 (31.4)	2027 (28.0)
Threatened or injured with a weapon on school property in the last 12 months			
No	12755 (92.1)	6000 (90.0	6755 (93.9)
Yes	1083 (7.9)	649 (10.0)	434 (6.1)
Videogame (hours/school day)			
≤ 2 hours	2385 (18.4)	1521 (23.7)	864 (12.9)
3+ hours	11472 (81.7)	5143 (76.3)	6329 (87.1)
Physical fighting on school property in the last 12 months			
No	11868 (86.5)	5391 (82.1)	6477 (91.2)
Yes	1838(13.5)	1178 (17.9)	660 (8.8)

Table 2 shows the results for factors associated with physical fighting. All the results were similar for both sexes except for the factors: Ethnicity (American Indian or Alaska Native, and Native Hawaii or other Pacific Islander), and watching videogames. In order to adjust among other factors for sex, a multivariate analysis was conducted. The results are shown in Table 3. Respondents aged 17 years or older were less likely to report physical fighting than those who were 14 years or younger (OR = 0.43; 95% CI [0.31, 0.59] for 17 years old and OR = 0.41; 95% CI [0.29, 0.59] for 18 years olds or older). Compared to Whites, American Indian or Alaska Native, Natives, Blacks, Native Hawaii or other Pacific Islanders, and Hispanics were more likely to report physical fighting on school property (OR = 2.11; 95% CI [1.22, 3.66], OR = 1.72; 95% CI [1.42, 2.09], OR = 2.18; 95% CI [1.01, 4.79], and OR = 1.74; 95% CI [1.41, 2.16] respectively). Physical fighting on school property was also positively associated with cigarette smoking (OR = 1.70; 95% CI [1.37, 2.10]), drinking alcohol (OR = 1.45; 95% CI [1.20, 1.76]), use of illegal drugs (OR = 1.73; 95% CI [1.42, 2.12]), having had property stolen or deliberately damaged on school property (OR = 2.06; 95% CI [1.74, 2.44]), having been threatened or injured with a weapon on school property (OR = 2.63; 95% CI [2.06, 3.34]), and playing videogame three or more hours a day (OR = 1.29; 95% CI [1.07, 1.56]).

**Table 2:** Results of bivariate stratified analysis on the association between physical fighting on school property and selected characteristics among US High School students, 2005

Characteristics	Total	Males	Females
Age (years)	Unadjusted OR [95% CI]		
≤ 14	1.00	1.00	1.00
15	1.09 [0.86, 1.37]	1.31 [0.96, 1.78]	0.79 [0.55, 1.14]
16	0.92 [0.73, 1.16]	0.99 [0.73, 1.35]	0.71 [0.49, 1.04]
17	0.59 [0.46, 0.75]	0.70 [0.51, 0.96]	0.40 [0.27, 0.59]
18+	0.61 [0.46, 0.80]	0.64 [0.45, 0.91]	0.46 [0.29, 0.75]
Gender			
Females	1.00		
Males	2.31 [2.01, 2.64]	-	-
Ethnicity			
White	1.00	1.00	1.00
American Indian or Alaska Native	2.15 [1.34, 3.43]	2.49 [1.44, 4.31]	1.83 [0.70, 4.77]
Asians	0.68 [0.38, 1.08]	0.68 [0.34, 1.12]	0.68 [0.39, 1.19]
Blacks	1.54 [1.32, 1.80]	1.30 [1.06, 1.59]	2.19 [1.69, 2.83]
Native Hawaii or other Pacific Islander	2.46 [1.26, 4.82]	3.17 [1.28, 7.86]	2.42 [0.90, 6.46]
Hispanics	1.53 [1.27, 1.84]	1.46 [1.15, 1.85]	1.80 [1.32, 2.45]
Cigarette smoking in the last 30 days			
No	1.00	1.00	1.00
Yes	2.52 [2.19, 2.91]	2.38 [1.99, 2.86]	2.95 [2.32, 3.74]
Alcohol use in the last 30 days			
No	1.00	1.00	1.00
Yes	2.12 [1.85, 2.44]	2.24 [1.88, 2.66]	1.98 [1.58, 2.49]
Ever used illegal drugs			
No	1.00	1.00	1.00
Yes	2.72 [2.37, 3.13]	2.44 [2.04, 2.90]	3.33 [2.62, 4.24]
Property stolen or deliberately damaged on school property in the last 12 months			
No	1.00	1.00	1.00
Yes	2.51 [2.20, 2.86]	2.30 [1.95, 2.72]	2.85 [2.28, 3.57]
Threatened or injured with a weapon on school property in the last 12 months			
No	1.00	1.00	1.00
Yes	4.68 [3.74, 5.84]	4.72 [3.96, 5.63]	4.22 [3.15, 5.64]
Videogame (hours/school day)			
≤ 2	1.00	1.00	1.00
3+	1.44 [1.20, 1.87]	1.60 [1.37, 1.87]	1.31 [0.97, 1.77]

**Table 3:** Multivariate analysis results on the association between physical fighting on school property and selected characteristics among US High School students, 2005

Characteristics	Adjusted OR [95% CI]
Age (years)	
≤ 14	1.00
15	0.91 [0.67, 1.24]
16	0.74 [0.54, 1.01]
17	0.43 [0.31, 0.59]
18+	0.41 [0.29, 0.59]
Gender	
Female	1.00
Male	2.23 [1.89, 2.63]
Ethnicity	
White	1.00
American Indian or Alaska Native	2.11 [1.22, 3.66]
Asians	0.70 [0.38, 1.28]
Blacks	1.72 [1.42, 2.09]
Native Hawaii or other Pacific Islander	2.18 [1.01, 4.79]
Hispanics	1.74 [1.41, 2.16]
Cigarette smoking in the last 30 days	
No	1.00
Yes	1.70 [1.37, 2.10]
Alcohol use in the last 30 days	
No	1.00
Yes	1.45 [1.20, 1.76]
Ever used illegal drugs	
No	1.00
Yes	1.73 [1.42, 2.12]
Property stolen or deliberately damaged on school property in the last 12 months	
No	1.00
Yes	2.06 [1.74, 2.44]
Threatened or injured with a weapon on school property in the last 12 months	
No	1.00
Yes	2.63 [2.06, 3.34]
Video game (hours/school day)	
≤ 2	1.00
3+	1.29 [1.07, 1.56]

## Discussion

Using data from the United States Youth Risk Behavior Survey 2005, we have estimated the 12 months prevalence of having engaged in a physical fight on school property among US adolescents in high school at 13.5%. Males were more likely to be involved in physical fighting than females (18.2% versus 8.8%). The prevalence of other relevant behavioral experiences were as follows: current smoking (23%); alcohol (43.3%); lifetime illicit drug use (44%); property damaged or stolen on school grounds (29.7%) and having been threatened or injured with a weapon (7.9%). All these prevalence estimates suggest that US adolescent experience unhealthy lifestyles including being stressed from external sources to a great degree.

In both bivariate and multivariate analysis the following were positively associated with having engaged in a physical fight on school property: male gender; non-white race (except Asians); substance use (alcohol, cigarette, and illicit drugs); having had property stolen or damaged; having ever been threatened or injured with weapon and exposure to video games.

The higher male prevalence compared to females is perhaps not surprising. Males top females in almost all unhealthy behaviors such as homicides, alcohol, cigarette smoking, not presenting for health care when ill and having multiple and concurrent sex partners [16-21]. Cultural perceptions of masculinity are likely to be the reasons that males predominate in many unhealthy behaviors [22,23]. Society is likely to be tolerant to male violence behaviors than it is to female violent behaviors. As male adolescents may feel violence among males is acceptable, there may be no strong disincentives to prevent them from violent behaviors.

We have observed the race-ethnicity differences that are associated with physical fighting among high school students in this study. Compared to Whites, American Indian or Alaska Native, Blacks and Hispanics were more likely to report physical fighting while Asians were less likely to engage in physical fighting. We do not believe these differences are due to any genetic differences but rather as a result of different socio-economic environments that adolescents live in. Previous studies have reported that with the exception of Asians, members of all other ethnic minority groups were more socioeconomically disadvantaged than White and that low socioeconomic status (SES) was a determinant of poor health outcomes [24-26]. Racial difference in health indices such as cardiovascular, perinatal and breast cancer mortality have consistently been observed [27-29], largely as a result of differences in access to health care. Furthermore almost always, with regarding to violence and incarcerations, Blacks and Hispanics are more likely to experience these than white. Ethnic minority youth live in unsafe environments, and so are exposed to violence and crime more than whites. As a result of this influence, they are also likely to engage in violent behaviors.

Adolescents who reported having had property damaged or stolen at school and those who reported having been threatened or injured with a weapon were more likely to have engaged in a fight. This may suggest that previous victimization may trigger adolescents to engage in a fight rather than solve interpersonal differences in a different way. Rudatsikira et al have reported that weapon carrying by California adolescents was associated with previous victimization [30]. These authors suggested that adolescents might have carried weapons in self-defense for any potential victimization. Glew et al have reported that victims of bullying in a school district in the United States felt it was "not wrong" to carry weapons to schools [31]. Among this group of 5391 school adolescents, 26% had been involved in bullying either as victims or as perpetrators.

We found that adolescents less than 14 years were more likely to report having engaged in a fight than older study participants. We believe this may be due to the fact that younger adolescents may not have gotten non-violent conflict resolution skills compared to older adolescents. Furthermore, younger adolescents are less likely to severely injured others such that the consequences of their actions attract parental or school authorities attention. Finally, parents are likely to neglect young adolescents fighting than if fighting occurs among older adolescents. All these reasons may discourage fighting among older adolescents while younger adolescents may not have as much disincentive. Johnson et al [32] have reported a qualitative study in which via focus group discussions, youth reported that fighting may be a necessary outcome of a disagreement. Although it would obviously be desirable that no physical fighting occurred, the youths interviewed suggested that physical fighting had potential to prevent more serious violence.

The association between self reported history of having 3 or more hours of video-game play and physical fighting is interesting. Ez-Elarab et al [33] have reported that elementary school children who had preference of violent video games were more likely to exhibit violent behaviors than those who did not have that preference. Previous reports have suggested that short-term effects of exposure to media violence are mostly due to the following: i) priming processes, ii) arousal processes, and ii) immediate mimicking of specific behaviors [34-36]. In brief, the priming process is when watching violent media makes the individual to associate similar situation seen in the video but now in real life to violence. Arousal is the process where having viewed violent media makes one excited and may engage in violence when provoked. Mimicry occurs when imitation occurs as part of observational learning [37-39].

The association between the numbers of hours adolescents play video games and having been engaged in a physical fight may also be just a marker of unsupervised time that the adolescent has at his or her disposal. Adolescents who are alone at home or elsewhere may be more likely to play video games than those that are supervised. Unsupervised adolescents are more likely to engage in a diversity of anti-social and unhealthy life styles such as sexual intercourse, cigarette smoking, violence and illicit drug use [40-43].

### Limitations of the study

This study has several limitations. This is a cross-sectional study so the results cannot be used to assume causality. The study participants may have underreported or over-reported on any of risk behaviors. The study methodology however has demonstrated good test-retest reliability [13]. Ethno-racial categories were based on self-categorized status. Mixed race adolescents may not have known that official racial categorization may be different from their own perceptions. An example is an mixed race adolescent with one parent black and the other white. The official categorization of such an individual is black despite the fact that some adolescents in that category may classify themselves as white. Intrinsically of course, there is no reason as to why such an adolescent should be classified as one and not the other.

The study recruited adolescents in school. These findings may therefore not be representative of out of school adolescents. In 2001, approximately 5% of persons aged 16 – 17 years in the United States were not enrolled in a high school program and had not completed high school [12]. We believe the proportion of out of school but age eligible adolescents to be relatively small.

Furthermore, the study did not categories the gravity or seriousness of the fighting. This therefore has meant that students who were involved in minor incidents have been categorized together with those where significant fighting may have occurred, such as those leading to hospitalization.

## Conclusion

These findings suggest that physical fighting among US High School students is widespread and positively associated with victimization (having been threatened or injured with a weapon on school property) and other risky behaviors such as smoking, alcohol and drugs use. Intervention programs to prevent/control those risky behaviors as well as further attention on the association between physical fighting and victimization at school through longitudinal research are warranted.

## Competing interests

The authors declare that they have no competing interests.

## Authors' contributions

ER conceived data analysis plan, conducted data analysis, and participated in the drafting of the manuscript.

ASM participated in the interpretation of the results and drafting of the manuscript.

SS participated in the interpretation of the results and drafting of the manuscript.

All authors read and approved the manuscript.

## References

[B1] Centers for Disease Control and Prevention School-associated homicides – United States, 1992–2006 MMWR Morb Mortal Wkly Rep 2008 57 2 33 36 18199965

[B2] Centers for Disease Control and Prevention (CDC) Source of firearms used by students in school-associated violent deaths – United States, 1992–1999 MMWR Morb Mortal Wkly Rep 2003 52 9 169 172 12650628

[B3] Kachur SP Stennies GM Powell KE Modzeleski W Stephens R Murphy R Kresnow M Sleet D Lowry R School-associated violent deaths in the United States, 1992 to 1994 JAMA 1996 275 1729 1733 10.1001/jama.275.22.1729 8637169

[B4] Pickett W Craig W Harel Y Cunningham J Simpson K Molcho M Mazur J Dostaler S Overpeck MD Currie CE HBSC Violence and Injuries Writing Group. Cross-national study of fighting and weapon carrying as determinants of adolescent injury Pediatrics 2005 116 e855 863 10.1542/peds.2005-0607 16322143

[B5] Rudatsikira E Siziya S Kazembe LN Muula AS Prevalence and associated factors of physical fighting among school-going adolescents in Namibia Ann Gen Psychiatry 2007 6 18 17650328 10.1186/1744-859X-6-18 PMC1947983

[B6] Moskowitz H Griffith JL DiScala C Sege RD Serious injuries and deaths of adolescent girls resulting from interpersonal violence: characteristics and trends from the United States, 1989–1998 Arch Pediatr Adolesc Med 2001 155 903 908 11483117 10.1001/archpedi.155.8.903

[B7] Miller TR Pindus N Douglass J Rossman S Databook on Nonfatal Injury – Incidence, Costs, and Consequences 1995 Washington, DC: Urban Institute

[B8] Moskowitz H Griffith JL DiScala C Sege RD Serious injuries and deaths of adolescent girls resulting from interpersonal violence: characteristics and trends from the United States, 1989–1998 Arch Pediatr Adolesc Med 2001 155 903 908 11483117 10.1001/archpedi.155.8.903

[B9] Centers for Disease Control and Prevention (CDC) Violence-related behaviors among high school students – United States, 1991–2003 MMWR Morb Mortal Wkly Rep 2004 53 29 651 655 15282447

[B10] Kodjo CM Auinger P Ryan SA Prevalence of, and factors associated with, adolescent physical fighting while under the influence of alcohol or drugs J Adolesc Health 2004 35 e11 6 15830460

[B11] Quality Education Data Products and services 2004 Denver, CO: Quality. Education Data, Inc

[B12] US Department of Education, National Center for Education Statistics. Common Core of Data Public Elementary/Secondary School Universe Survey: School Year 2002–03 2004 Washington, DC: US Department of Education, National Center for Education Statistics http://nces.ed.gov/ccd Retrieved on 5 January 2008

[B13] Brener ND Kann L McManus T Kinchen SA Sundberg EC Ross JG Reliability of the 1999 Youth Risk Behavior Survey questionnaire J Adolesc Health 2002 31 336 342 10.1016/S1054-139X(02)00339-7 12359379

[B14] Hill SC Drolet JC School-related violence among high school students in the United States, 1993–1995 J Sch Health 1999 69 264 272 10529964 10.1111/j.1746-1561.1999.tb06406.x

[B15] Orpinas PK Basen-Engquist K Grunbaum JA Parcel GS The co-morbidity of violence-related behaviors with health-risk behaviors in a population of high school students J Adolesc Health 1995 16 216 225 10.1016/1054-139X(94)00067-O 7779832

[B16] Dobson R Danish men consult GPs less than women but attend hospital more and have greater mortality BMJ 2007 335 1010 1011 18006979 10.1136/bmj.39395.720995.DB PMC2078631

[B17] Siziya S Muula AS Kazembe LN Rudatsikira E Harmful lifestyles' clustering among sexually active in-school adolescents in Zambia BMC Pediatr 2008 8 6 18267020 10.1186/1471-2431-8-6 PMC2276492

[B18] Rudatsikira E Ogwell AE Siziya S Muula AS Prevalence of sexual intercourse among school-going adolescents in Coast Province, Kenya Tanzan Health Res Bull 2007 9 159 163 18087892 10.4314/thrb.v9i3.14322

[B19] Smith DJ Modern marriage, men's extramarital sex, and HIV risk in southeastern Nigeria Am J Public Health 2007 97 997 1000 10.2105/AJPH.2006.088583 17463366 PMC1874209

[B20] Mahalik JR Burns SM Syzdek M Masculinity and perceived normative health behaviors as predictors of men's health behaviors Soc Sci Med 2007 64 2201 2209 10.1016/j.socscimed.2007.02.035 17383784

[B21] Courtenay WH Constructions of masculinity and their influence on men's well-being: a theory of gender and health Soc Sci Med 2000 50 1385 1401 10.1016/S0277-9536(99)00390-1 10741575

[B22] Ng N Weinehall L Ohman A 'If I don't smoke, I'm not a real man' Indonesian teenage boys' views about smoking Health Educ Res 2007 22 794 804 10.1093/her/cyl104 16987943

[B23] Hunt K Hannah MK West P Contextualizing smoking: masculinity, femininity and class differences in smoking in men and women from three generations in the west of Scotland Health Educ Res 2004 19 239 249 10.1093/her/cyg061 15140844

[B24] Sudano JJ Baker DW Explaining US racial/ethnic disparities in health declines and mortality in late middle age: The roles of socioeconomic status, health behaviors, and health insurance Soc Sci Med 2006 62 4 909 922 10.1016/j.socscimed.2005.06.041 16055252

[B25] Mendes de Leon Glass CF Anderson NB, Bulatao RA, Cohen B The role of social and personal resources in ethnic disparities in late-life health Critical perspectives on racial and ethnic differences in health in late life 2004 The National Academies Press, Washington, DC 353 405 20669464

[B26] Williams DR Collins C Racial residential segregation: a fundamental cause of racial disparities in health Public Health Rep 2001 116 404 416 12042604 10.1093/phr/116.5.404 PMC1497358

[B27] Polednak AP Racial differences in mortality from obesity-related chronic diseases in US women diagnosed with breast cancer Ethn Dis 2004 14 463 468 15724763

[B28] Curtis E Quale C Haggstrom D Smith-Bindman R Racial and ethnic differences in breast cancer survival: how much is explained by screening, tumor severity, biology, treatment, comorbidities, and demographics? Cancer 2008 112 171 180 10.1002/cncr.23131 18040998 PMC2674622

[B29] Wingate MS Alexander GR Racial and ethnic differences in perinatal mortality: the role of fetal death Ann Epidemiol 2006 16 485 491 10.1016/j.annepidem.2005.04.001 15993623

[B30] Rudatsikira E Singh P Job J Knutsen S Variables associated with weapon-carrying among young adolescents in southern California J Adolesc Health 2007 40 470 473 10.1016/j.jadohealth.2006.12.011 17448409

[B31] Glew GM Fan MY Katon W Rivara FP Bullying and school safety J Pediatr 2008 152 123 128 10.1016/j.jpeds.2007.05.045 18154913 PMC3839286

[B32] Johnson SB Frattaroli S Wright JL Pearson-Fields CB Cheng TL Urban youths' perspectives on violence and the necessity of fighting Inj Prev 2004 10 287 291 15470008 10.1136/ip.2004.005793 PMC1730136

[B33] Ez-Elarab HS Sabbour SM Gadallah MA Asaad TA Prevalence and risk factors of violence among elementary school children in Cairo J Egypt Public Health Assoc 2007 82 127 146 18217328

[B34] Huesmann LR Kirwil L Flannery D Why observing violence increases the risk of violent behavior in the observer The Cambridge Handbook of Violent Behavior and Aggression 2007 Cambridge: Cambridge University Press

[B35] Berkowitz L LePage A Weapons as aggression-eliciting stimuli J Pers Soc Psychol 1967 7 202 207 10.1037/h0025008

[B36] Bryant J Zillmann D Effect of intensification of annoyance through unrelated residual excitation on substantially delayed hostile behavior J Exp Soc Psychol 1979 15 470 480 10.1016/0022-1031(79)90009-X

[B37] Geen RG O'Neal EC Activation of cue-elicited aggression by general arousal J Pers Soc Psychol 1969 11 289 292 10.1037/h0026885 5784269

[B38] Huesmann LR Hurley S, Chater N Imitation and the effects of observing media violence on behavior Perspectives on Imitation: From Neuroscience to Social Science: Imitation, Human Development, and Culture 2005 2 Cambridge, Massachusetts: MIT Press

[B39] Meltzoff AN Moore MK Muir D, Slater A Imitation of facial and manual gestures by human neonates: Resolving the debate about early imitation Infant Development: The Essential Readings 2000 Malden, Massachusetts: Blackwell Publishers

[B40] Mulhall PF Stone D Stone B Home alone: is it a risk factor for middle school youth and drug use? J Drug Educ 1996 26 39 48 8991968 10.2190/HJB5-0X30-0RH5-764A

[B41] Richardson JL Radziszewska B Dent CW Flay BR Relationship between after-school care of adolescents and substance use, risk taking, depressed mood, and academic achievement Pediatrics 1993 92 32 38 8516082

[B42] Coley RL Morris JE Hernandez D Out-of-school care and problem behavior trajectories among low-income adolescents: individual, family, and neighborhood characteristics as added risks Child Dev 2004 75 948 965 10.1111/j.1467-8624.2004.00716.x 15144496

[B43] Cohen DA Farley TA Taylor SN Martin DH Schuster MA When and where do youths have sex? The potential role of adult supervision Pediatrics 2002 110 e66 10.1542/peds.110.6.e66 12456933

